# Predictors of Mortality in Patients Undergoing Mitral Valve Replacement

**DOI:** 10.5539/gjhs.v8n3p37

**Published:** 2015-06-25

**Authors:** Muhammad Farhan Khan, Muhammad Shahzeb Khan, Faizan Imran Bawany, Mudassir Iqbal Dar, Mehwish Hussain, Saima Farhan, Kaneez Fatima, Khizar Hamid, Mohammad Hussham Arshad, Maira Aziz, Uswah Siddiqi, Nashit Irfan Aziz, Muhammad Bazil Musharraf, Abdul Bari Khan

**Affiliations:** 1 Civil Hospital, Karachi, Pakistan; 2 Dow Medical College, Dow University of Health Sciences, Karachi, Pakistan; 3 Aga Khan University Hospital, Karachi, Pakistan; 4 Department of Biological Sciences, The Lyceum; 5 Karachi Grammar School, Karachi, Pakistan

**Keywords:** mitral valve replacement, mortality, Pakistan, predictors

## Abstract

**Objective:**

Although mitral valve replacement is frequently performed in patients of all age groups, there are few studies available which determine the causes of operative mortality in mitral valve replacement especially in our region. Therefore, the objective of this study was to identify factors that are significantly associated with operative mortality in mitral valve replacement.

**Methods:**

From August 2012 to March 2013, 80 consecutive patients undergoing mitral valve replacement in a single tertiary hospital were included. Patients with a history of previous coronary artery bypass graft surgery or congenital heart problems were excluded from the sample. The included patients were observed for a period of 30 days. Pre and post-operative variables were used to identify significant predictors of mortality.

**Results:**

The overall hospital mortality (30 days) was 15%. High post-perative creatinine (P =0.05), high ASO titre (P=0.03), young age (P=0.011), low cardiac output (P=0.0001), small mitral valve size (P=0.002) and new onset of atrial fibrillation (P=0.007) were the significant independent predictors of operative morality.

**Conclusion:**

Mitral valve replacement can be performed in third world countries with limited resources with low mortality. However, optimal selection of mitral valve size can help to improve operative mortality.

## 1. Introduction

Mitral valve pathology is the most common disorder amongst all valvular heart diseases. Though symptomatic improvement may be attained through medical treatment, many patients require minimally invasive procedures or replacement of the diseased valve for improvement of their condition (Turi, 2004). Sudden- onset (acute) cases of mitral regurgitation usually arise due to infection of mitral valve or abrupt disruption of valves that may be due to rupture of one of the supporting muscles following ischemic damage due to myocardial infarction. Many acute mitral regurgitation patients are in a critical condition and should be indicated for emergent mitral valve replacement.

The main idea behind surgical valve replacement in valvular heart disease is that the prosthesis selected to substitute for a stenotic or regurgitant valve will result in improvement in cardiac function, keeping in mind the complications associated with surgery and mechanical prosthetic valves (Bolman, 2007). Although not universal, it is a well-known fact that replacement of mitral valve is associated with greater short term and long term mortality as compared to mitral valve repair (Suri et al., 2006). Therefore, mitral valve repair is a more common surgical modality opted for, followed by mitral valve replacement (Turi, 2004). As a result, it is important to determine which preoperative and operative variables are associated with increased operative 30 day mortality following mitral valve replacement. We hypothesize that certain variables such as gender, age, Left Ventricular End Systolic Dimension (LVESD), low cardiac output and size of the prosthetic mitral valve may have a significant impact on operative mortality.

Therefore, the purpose of our study was to identify variables that are significantly associated with 30 day operative mortality in mitral valve replacement.

## 2. Methodology

In this prospective study, 80 consecutive patients undergoing mitral valve replacement in a single tertiary hospital were included. The data was collected during the time period of 7 months from August 2012 to March 2013. All patients with mitral valve disease such as stenosis or regurgitation were a part of our study. However, patients with a history of previous coronary artery bypass graft surgery or congenital heart problems were excluded from the sample. Similarly, patients who were undergoing concomitant surgical procedures such as aortic valve replacement or coronary artery bypass graft surgery were also excluded from the study. The included patients were observed for a period of 30 days. Pre-operative profile and operative and post-operative variables were gathered for the sample. Pre-operative profile included variables such as age, gender, blood group, Anti Streptolysin O (ASO) titre and New York Heart Association (NYHA) classification. Moreover, left ventricular function details and mitral valve areas were noted after the echocardiogram. The operative variables such as cross clamp and bypass time were jotted down by the surgeon himself.

All mortalities that occurred during the hospital stay and those that occurred after discharge but within 30 days of the valve replacement were defined as operative mortality. However, deaths which had no association with the operation were not included in operative mortalities. The pre and post-operativevariables were used to identify significant predictors ofoperative mortality in mitral valve replacement. Informed written consent was taken from each patient. For entering and analysis of data, SPSS version 21 was used. Mean ± standard deviation was computed for continuous variables. Frequency and percentages were calculated for categorical variables. Mann-whitney U test and chi-square with Fisher's exact test were run to compare outputs in expired and alive patients. P value less than 0.05 was considered as significant.

## 3. Results

The incidence of 30 days mortality in mitral valve replacement was found to be 15% (n=12). The mean age of the patients was 30 ± 11 years. More than of the patients were male (n = 46, 57.5%). O +ve (n = 34, 42.5%) and B +ve (n = 27, 33.8%) blood groups were more common among the patients. Only one (1.3%) patient had a previous history of myocardial infarction while 2 (2.5%) patients were obese. Fifty five (68.8%) patients were classified as NYHA class 1 and 2 whereas the remaining patients (31.3%) were classified as class 3 and 4. Hepatitis profile of 10 (12.5%) patients was positive (Table 1).

**Table 1:** Showing the baseline variables of the patients

Mean Age (years)		30 ± 11
Gender	male	34 (42.5%)
	female	46 (57.5%)
Blood group	A+ve	9 (11.3%)
	A-ve	2 (2.5%)
	B+ve	27 (33.8%)
	B-ve	1 (1.3%)
	O+ve	34 (42.5%)
	O-ve	1 (1.3%)
	AB+ve	5 (6.3%)
	AB-ve	1 (1.3%)
History of myocardial infarction	yes	1 (1.3%)
	no	79 (98.8%)
Obesity	yes	2 (2.5%)
	no	78 (97.5%)
NYHA classification	class 1, class 2	55 (68.8%)
	class 3, class 4	25 (31.3%)
Hepatitis profile	negative	70 (87.5%)
	positive	10 (12.5%)

The patients who expired were significantly younger than those who survived (P = 0.011). The NYHA classification was similar in both expired and alive patients (Table 2). The Anti Streptolysin O (ASO) titre of the patients was divided into groups, values less than 200 and more than 200. Fourteen patients had ASO titre greater than 200 and out of them 5 (35.7%) expired. Out of 66 patients who had ASO titre less than 200, 59 (89.4%) did not have operative mortality. This difference was statistically significant (P = 0.03). Atrial fibrillation was significantly more common in patients who expired as compared to those who survived (P = 0.007). Moreover, out of the 12 patients who died, 9 had low cardiac output (P < 0.0001). The postoperative creatinine value was on average two times higher in expired patients as compared to those who survived (P = 0.05) (Table 3).

**Table 2:** Comparison of baseline variables with operative mortality

		Expired	Alive	P Value
Mean Age (years)		24 ± 17	31 ± 10	0.011
Gender	male	6 (17.65)	28 (82.4%)	0.569
	female	6 (13%)	40 (87%)	
History of myocardial infarction	yes	0 (0%)	1 (100%)	>0.999
	no	12 (15.8%)	67 (84.8%)	
Obesity	yes	0 (0%)	2 (100%)	>0.999
	no	12 (15.4%)	66 (84.6%)	
NYHA classification	class 1, class 2	7 (12.7%)	48 (87.3%)	0.502
	class 3, class 4	5 (20%)	20 (80%)	

**Table 3:** Showing the predictors of mortality

		Expired	Alive	P Value
ASO titre	<200	7 (10.6%)	59 (89.4%)	0.03
	>200	5 (35.7%)	9 (64.3%)	
Atrial fibrillation	yes	8 (32%)	17 (68%)	0.007
	no	4 (7.3%)	51 (92.7%)	
Need for synchronized shock	yes	8 (61.5%)	5 (38.5%)	<0.0001
	no	4 (6%)	63 (94%)	
Low cardiac output	yes	9 (64.3%)	5 (35.7%)	<0.0001
	no	3 (4.5%)	63 (95.5%)
Post op creatinine		2.18 ± 2.07	0.94 ± 0.23	0.05

The mean size of the prosthetic mitral valve used was significantly smaller in expired patients as compared to those who did not suffer death (P = 0.002). However, mitral valve area and type of mitral valve disease (stenosis or regurgitation) were not significantly different in expired and alive patients. Intra aortic balloon pump was used in 4 patients and all of these 4 expired. Moreover, all the patients who survived did not require intra aortic balloon pump (P < 0.0001) (Table 4).

**Table 4:** Effect of operative variables on operative mortality

		Expired	Alive	P Value
left ventricular function	good	3 (8.8%)	31 (91.2%)	0.215*
	moderate	9 (20%)	36 (80%)	
	poor	0 (0%)	1 (100%)	
Mitral regurgitation	absent	5 (12.5%)	35 (87.5%)	0.755
	present	7 (17.5%)	33 (82.5%)	
Mitral stenosis	absent	5 (17.2%)	24 (82.8%)	0.749
	present	7 (15.6%)	44 (86.27%)	
Intra-aortic balloon pump	yes	4 (100%)	0 (0%)	<0.0001
	no	8 (10.5%)	68 (89.5%)	
Reopened	yes	4 (66.7%)	2 (33.3%)	0.004
	no	8 (10.8%)	66 (89.2%)	
Wound infection	yes	0 (0%)	2 (100%)	>0.999
	no	12 (15.4%)	66 (84.6%)
Mitral valve area		1.2 ± 0.5	1.1 ± 0.5	0.978
Pulmonary artery pressure		60 ± 14	63 ± 18	0.62
Systolic dimension (mm)		37 ± 12	36 ± 11	0.822
Diastolic dimension (mm)		52 ± 16	51 ± 12	0.736
Cross clamp time (minutes)		65 ± 39	57 ± 37	0.438
Bypass time (minutes)		98 ± 51	75 ± 47	0.088
Mitral valve size (mm)		27 ± 3	30 ± 2	0.002

* The p-value obtained by Mann-Whitney U test.

## 4. Discussion

The most serious consequence of rheumatic fever is rheumatic heart disease that occurs in roughly 30% of patients. Patients with sudden onset rheumatic fever may present with pancarditis along with valvular pathology, heart failure and pericarditis (Zakkar, Amirak, Chan, & Punjabi, 2009). Incidence of rheumatic heart disease has dropped during the last 40 years in western world. However, it is still remains a major health challenge in developing countries. It is estimated that 16 million individuals are affected by rheumatic heart disease around the globe, with roughly 281,000 new cases and 234,000 deaths every year (Carapetis, Steer, Mulholland, & Weber, 2005). One of the most common valvular complications of rheumatic heart disease is mitral stenosis and/or regurgitation. In majority of cases, mitral valve replacement is typically essential, however in few cases mitral valve repair can also be performed (Zakkar et al., 2009). Previous studies have shown that mitral valve replacement is associated with greater incidence of operative mortality (Suri et al., 2006). Therefore, our study highlights important pre and postoperative variables that could have a significant impact on operative mortality in mitral valve replacement.

Magnanti et al. (2010) and his colleagues have showed in their study that low cardiac output syndrome, after mitral valve surgery, significantly increases mortality and morbidity. The syndrome is characterized by the need of a post-operative intra-aortic balloon pump or inotropic support for longer than thirty minutes in intensive care unit. Therefore, mechanical circulatory support is required especially in high risk patients, in whom mitral valve surgery is becoming increasingly popular. Increased mortality due to low cardiac output after mitral valve surgery is also evident from the results of our study that show a significant association between the two (p<0.0001). However, the prevalence (17.5%) of low cardiac output syndrome was relatively greater in our study as compared to the prevalence of 7% reported by a previous study (Maganti et al., 2010).

The results of our study also indicate that small size of mitral valve was significantly associated with higher rates of mortality. This could be explained by patient prosthesis mismatch (PPM) after mitral valve replacement. It is believed that this mismatch can result in elevated transvalvular gradients mimicking those with mitral stenosis (Suri et al., 2006). Furthermore, these high transvalvular gradients can result in left atrium dilation which can subsequently cause atrial fibrillation. Magne et al. (2007) hypothesized that PPM might have significant impact on mortality after MVR. Our data also indicates that almost 25% of the patients had atrial fibrillation. The mechanism hypothesized above may be a crucial mechanism by which atrial fibrillation occurs after MVR. Hence optimal selection of prosthetic mitral valve size is essential. Optimal selection of prosthetic valve would lower down the chances of PPM greatly and consequently reduce mortality rates. Great implantability of the prosthetic valve can only be achieved be choosing an appropriate size of the valve being implanted.

One of the most common ways to assess kidney function is to measure serum creatinine levels. Serum creatinine levels remain mostly constant in patients with normal kidney function, with a daily fluctuation of roughly 7% (Traynor, Mactier, Geddes, & Fox, 2006). Our results show that post-operative creatinine had a significant impact on operative mortality in mitral valve replacement (P value=0.05). Magnanti et al. (2010) has also shown that decreased renal function is an independent predictor of mortality (odds ratio=4.3). Therefore, it can easily be inferred from these findings that proper renal function with adequate creatinine clearance can significantly lower mortality after mitral valve surgery. The association of increased mortality with elevated creatinine and NYHA class IV is further emphasized by the results of Nowicki et al and his colleagues (2004). Their results were supported by a high sensitivity and specificity index as measured through the area under receiver operating characteristic (ROC) curve (0.79, 95% CI, 0.76-0.81). In contrast, our results did not show a significant effect of NHYA class on 30 day mortality.

Moreover, the results of our study show that LVESD was not significantly different between expired and non-expired patients. In fact, the mean value of LVESD was almost identical in alive and expired patients. This is in contrast with a study that showed LVESD was a significant independent predictor of mortality in mitral valve replacement (Tribouilloy et al., 2009). However, they had only taken mitral regurgitation patients due to fail leaflets. In that study it was concluded that all patients should be operated before LVESD reaches 40 mm to reduce mortality. Our study did not show any such association and most of the patients who died had LVESD less than 40 mm.

Although our study has provided some interesting findings, there are a few limitations to that should be considered. Firstly, the small sample size of 80 patients makes it difficult to generalize all the findings obtained by the study. Secondly, we did not determine the predictors associated with mid or long term mortality. Studies in the future should focus on the significant predictors of long term mortality after mitral valve replacement by doing a long follow up. Additionally, health related quality of life should also be accessed after mitral valve replacement. Nevertheless, this study has listed down some of the significant predictors of mortality after mitral valve replacement in developing countries. It has also enlightened on the crucial issue of the selecting optimum size of the valve for which the researchers could also design new studies focusing on factors affecting the size of prosthetic mitral valve. These studies will help to lower down the PPM mismatch.

## 5. Conclusion

The results of our study indicate that young age, ASO titre greater than 200, postoperative atrial fibrillation, high post-operative creatinine levels, small prosthetic mitral valve size implanted and low cardiac output were significant predictors of mortality. We also conclude that small mitral valve size can lead to severe PPM and subsequently atrial fibrillation. This can be easily prevented by choosing prosthesis with a larger orifice area. However, more long term clinical studies with larger samples should be conducted to determine predictors of mortality after mitral valve replacement in developing countries.
